# Incidence of HIV infection and associated factors among men who have sex with men in Zhejiang, China: a cohort study

**DOI:** 10.3389/fpubh.2025.1551612

**Published:** 2025-04-28

**Authors:** Hui Wang, Wenkai Zhou, Xiaohong Pan, Qiaoqin Ma, Lin Chen, Xin Zhou, Tingting Jiang, Wanjun Chen

**Affiliations:** ^1^Zhejiang Provincial Center for Disease Control and Prevention, Hangzhou, China; ^2^Department of Epidemiology and Biostatistics, School of Public Health, Zhejiang University School of Medicine, Hangzhou, China

**Keywords:** HIV, sexual behavior, MSM, student, Zhejiang

## Abstract

**Aim:**

Men who have sex with men (MSM) contribute increasingly to the burden of HIV infection due to their high-risk sexual behaviors; however, studies focusing on the sexual behaviors among student MSM population remain limited. This study aims to investigate the incidence of HIV infection among MSM, while exploring high-risk sexual behavior in student MSM in Zhejiang province, China.

**Method:**

Prospective cohort study was conducted among MSM population in four cities in Zhejiang province. Information including socio-demographic characteristics as well as sexual behaviors were collected at baseline. Follow-up surveys and testing for HIV infection were conducted every 3 months. Univariable and multivariable logistic regression were performed to assess risk factors for HIV infection, investigate the sexual behavior differences between student and non-student MSM groups. Cox regression analyses were employed to discover potential association among various risk factors.

**Result:**

2081 HIV-negative MSM were enrolled in our cohort and 36 participants were infected with HIV. The incidence density of HIV infection was 2.15 per 100 person-years among this population. Student MSM are more likely to take sexual role of versatile (OR: 1.56, 95% CI: 1.05–2.32, *p* = 0.029) and receptive only (OR: 2.65, 95% CI: 1.62–4.08, *p* < 0.001) during anal intercourse, with a lower rate of previous HIV testing (OR: 0.45, 95% CI: 0.31–0.66, *p* < 0.001). Cox regression discovered that MSM who had more than 6 partners in anal sexual intercourse were more likely to obtain HIV-seroconversion than who had one fixed partner (HR: 5.14, 95% CI: 1.67–10.59, *p* < 0.001), participants who sometimes use condoms (HR: 4.11, 95% CI: 1.28–13.16, *p* = 0.017) and never use condoms (HR: 2.57, 95% CI: 1.16–5.66, *p* = 0.020) were more vulnerable to be infected by HIV compared to those use condoms constantly. Versatile (HR: 5.30, 95% CI: 1.65–17.06, *p* = 0.005) and receptive only role (HR: 3.23, 95% CI: 1.05–9.96, *p* = 0.042) in anal intercourse were more likely to be infected by HIV than insertive only.

**Conclusion:**

High-risk behaviors, particularly inconsistent condom uses during anal sex, significantly increase the risk of HIV infection. Student MSM exhibited persistent high-risk behaviors and low HIV testing rates. Greater attention and tailored interventions are needed to promote safer sexual practices and reduce HIV transmission in this population.

## Introduction

Human Immunodeficiency Virus (HIV)/acquired immunodeficiency syndrome (AIDS) continues to impose a significant burden on global health. According to the latest report from the Joint United Nations Program on HIV/AIDS (UNAIDS), there were 39.9 million people living with HIV globally in 2023, including 1.3 million new infections and 630,000 deaths due to AIDS-related diseases, with 29.8 million individuals receiving antiretroviral therapy (ART) (1). Despite significant reductions in Disability-Adjusted Life Years (DALYs) attributed to HIV/AIDS due to the availability of treatments like ART, HIV/AIDS remained one of the top ten leading causes of DALYs in many countries as of 2021 (2, 3). In recent years, the sharply increasing HIV prevalence among men who have sex with men (MSM) has drawn attention from public health authorities (4–7). In China, a meta-analysis of HIV prevalence among the MSM population from 2001 to 2018 found an average prevalence of 5.7%, with some cities reporting rates as high as 13.7% (8). Similarly, studies conducted during the same period reported HIV prevalence of 17.4% among MSM in Mexico (9), 17.9% in Sub-Saharan Africa, and 25.4% in Caribbean countries (10). The MSM population have contributed a lot in HIV/AIDS burden due to specific high-risk behaviors, such as engaging in both insertive and receptive (versatile) anal intercourse, unprotected anal and oral sex, having multiple sexual partners, and participating in commercial sexual activities (5, 11). Although MSM account for a disproportionate share of the HIV burden, there is still a lack of large-scale cohort studies in China examining the HIV incidence and associated high-risk behaviors within this group.

Historically, students were not considered a high-risk group for HIV infection (12). However, in recent years, the incidence of HIV among young students has increased significantly. A global burden of disease study highlighted that, as of 2019, HIV was the second leading cause of burden among adolescents and young adults aged 10–24 (13). Data from China on HIV-positive students revealed that 82.6% of new infections on campuses occurred among MSM, emphasizing the urgent need to explore the high-risk behaviors and unique characteristics of this population (14). Public health authorities have implemented various measures to curb HIV transmission in higher education institutions, such as organizing educational seminars and improving access to HIV testing. Despite the contribution of MSM to HIV transmission on campus, research on the high-risk sexual behaviors of student MSM remained insufficient, particularly studies focusing on the sexual roles during anal intercourse among this population. Addressing this gap is crucial for designing targeted interventions to combat the epidemic in this vulnerable population on campus.

Zhejiang Province, an economically developed region in eastern China, has a population of over 60 million, with a per capita disposable income of approximately 5,500 yuan per month. By October 2023, there were 42,100 reported cases of HIV infection in the province, resulting in 5,236 deaths due to HIV-related causes (15). To better understand the status of HIV infection and high-risk behaviors among men who have sex with men (MSM) in Zhejiang, we conducted a prospective cohort study. The study aimed to investigate the potential relationship between high-risk behaviors and HIV seroconversion, as well as to identify prevalent high-risk sexual behaviors within the student MSM population. The findings of this study will provide valuable insights into these high-risk behaviors and help inform targeted prevention strategies.

## Methods

### Study design

We conducted a prospective cohort of MSM population in Zhejiang province from March 2019. The data for this study was collected from March 2019 to March 2020, with an observation period of 1 year. Each participant was followed up every 3 months after recruitment, with reminders provided by members of a social organization through phone calls or WeChat messages to ensure consistent follow-up. The data of the current study originated from Prevention’s HIV and STD Prevention and Control Institute, Zhejiang Provincial Center for Disease Control. Participants had to make completion of the same questionnaire about their socio-demographic as well as sexual behavior information and a blood specimen for HIV will be extracted after finishing questionnaires. Both the questionnaire and blood samples of every participant were associated and identified by assigning a unique code to ensure that privacy will not be leaked. The study obtained informed consent from all participants.

### Participant and sampling methods

The inclusion criteria for the participants in the study were: (1) MSM; (2) 18 years of age and above; (3) Participants who tested negative for HIV upon entering the cohort (i.e., negative during the window period, defined as testing negative within 3 months of their most recent high-risk behavior should be excluded); (4) currently living in local area and (5) have willing to receive regular follow-up testing and surveys. Additionally, we divided the target population into student MSM and non-student MSM based on participants’ self-reported baseline occupation (student or non-student) to compare behavioral differences between the two groups. The Respondent-driven sampling (RDS) method was employed to recruit initial seeds on applications such as *Blued,* a social networking platform exclusively for the MSM community. Participants will be explained to the design of our study and potential risks that may occur in the procedure as well as the benefits that they may obtain after each follow-up.

### Questionnaire interview

The questionnaire involves two parts. Socio-demographic information of the participants was collected in the first part including age, ethnicity, level of education, monthly income, marital status while second part is the characteristics of sexual behaviors such as sexual orientation, whether condoms have been consistently used during anal intercourse, whether have had intercourse with male after drinking, whether have used condoms during heterosexual intercourse, whether have had an HIV testing before entering the cohort, number of male sexual partners, whether had group sex behavior, whether had HIV-positive sex partners, sexual roles in anal sex, whether had anal or oral intercourse with man. In both homosexual anal intercourse and heterosexual sexual behavior, the frequency of condom use is categorized as follows: “Frequently” encompasses both “Always” and “Often,” indicating consistent condom use as well as frequent use. “Sometimes” is defined as falling between “Never” and “Frequently.” For group sex behavior, “Sometimes” is defined as engaging in the behavior at least once a month, while “Seldom” is defined as once every 3 months.

### Laboratory testing

Participants will be asked to draw blood specimens by doctors from CDC in the local area for HIV and syphilis antibody detection before the interview. Enzyme Linked Immunosorbent Assay (ELISA) and rapid reverse transcription (RT) test will be carried out for HIV virus antibody detection. MSM with positive results were then confirmed by the Western blot assay (HIV Blot). Rapid Plasma Reagin (RPR) is suitable for screening syphilis antibodies, and participants who are positive in the *Treponema Pallidum* Particle Agglutination test (TPPA) are considered positive with syphilis. The results will be communicated to the participants by a doctor via phone.

### Statistical analysis

The questionnaire’s information was doubly entered. We described the socio-demographic characteristics and sexual behaviors of all the participants included. Categorical variables were performed by numbers and percentages while continuous variables were expressed in the form of mean ± standard deviation. The density of HIV incidence was calculated with person-years (PYs). The Chi-square and *t*-test were used to compare the behavioral characteristics between HIV positive and negative participants and a two-tailed *p* < 0.05 was considered statistically significant. Univariable and multivariable logistic regression were performed to evaluate risk factors for HIV infection, while multivariate logistic regression compared sexual behavior differences between student and non-student MSM groups. Both socio-demographic and sexual behavior factors with statistical significance in univariable analysis will be included in the multivariable logistic regression. All analyses were performed by IBM SPSS software version 25.0.

### Ethics approval

The study was approved by the ethical review board of Zhejiang Provincial Center for Disease Control and Prevention. The research presented no harm or risk to the participants, and their confidentiality and anonymity was strictly preserved. No personal identifying information was provided, all staff underwent confidentiality training, and the data was used solely for scientific research purposes. The study was carried out with the intention of improving the health of MSM in Zhejiang Province. All participants gave written informed consent during the survey.

## Result

### Baseline characteristics

Basic information of social-demographic and sexual behavior information was shown in Table 1. A total of 2,147 MSM were recruited during the entire study period and 2081 (96.9%) initially HIV-negative participants were enrolled in our cohort. The majority (1,563, 75.1%) of participants were under 35 and the median age was 27 years. 2000 (96.1%) participants belonged to the Han group, with 500 (24%) participants earning less than 3,000 yuan monthly. Nearly two-thirds (1,366, 65.6%) of the participants were single when enrolled in baseline screening. The details of baseline sexual behaviors were shown in Table 2. more than half (1,099, 52.8%) of participants were self-reported homosexual and 1768 (85%) have had anal or oral sex with male. 166 (9.3%) of them have not had sex with male in the past 6 months, and 818 (46.2%) have one fixed male sexual partner. Less than two thirds (966, 60.3%) of the participants who have had sex with male use condoms consistently during anal intercourse while 81 (5.0%) never use condom. 625 (41%) of participants who have had anal intercourse were versatile (take both insertive and receptive sexual roles), 839 (40.3%) used condoms during intercourse with females. Detailed baseline difference of student MSM (*n* = 214) and non-student MSM (*n* = 1867) were provided in Supplementary Table 1.

**Table 1:** Basic characteristics of MSM cohort in Zhejiang (*n* = 2081).

Variables	Category	*n* (%)
Age	18–24	724 (34.8)
	25–34	839 (40.3)
	35–44	333 (16.0)
	≥45	184 (8.9)
Ethnicity	Han	2000 (96.1)
	Minority	81 (3.9)
Area	Ningbo	1,110 (53.3)
	Jinhua	550 (26.4)
	Jiaxing	301 (14.5)
	Taizhou	120 (5.8)
Local residence time	Always	714 (34.3)
	2 years and above	551 (26.5)
	Less than 2 years	816 (39.2)
Education level	Postgraduate and above	68 (3.2)
	Undergraduate (College)	965 (46.3)
	High school (secondary school)	622 (29.9)
	Junior high school	351 (16.8)
	Elementary school and below	80 (3.8)
Monthly income (yuan)	No income	199 (9.5)
	≤1,500	81 (3.9)
	1,501–3,000	220 (10.6)
	3,001–5,000	704 (33.8)
	5,001–10,000	719 (34.6)
	10,001–50,000	137 (6.6)
	Above 50,000	21 (1.0)
Marital status	Single	1,366 (65.6)
	Married or cohabited	582 (28.0)
	Divorced or widowed	133 (6.4)
Sexual orientation	Homosexual	1,099 (52.8)
	Bisexual	590 (28.3)
	heterosexual	89 (4.3)
	Uncertain	303 (14.6)
Whether had anal or oral intercourse with man	Yes	1768 (85.0)
	No	313 (15.0)
Number of male sexual partners in the past 6 months (*n* = 1768)	None	166 (9.3)
	1	818 (46.2)
	2–5	677 (38.3)
	6–9	61 (3.6)
	10 and above	46 (2.6)
Whether had HIV-positive sex partners in the past 6 months (*n* = 1,654)	None	1,009 (61.0)
	Have an HIV-positive sex partner and have been receiving antiviral treatment	31 (1.8)
	Have an HIV-positive sex partner but have not been receiving antiviral treatment	8 (0.5)
	Not clear	606 (36.7)
Whether to use a condom during anal intercourse in the past 6 months (*n* = 1,602)	Always	966 (60.3)
	Sometimes	478 (29.8)
	Never	81 (5.0)
	No anal intercourse	77 (4.8)
Sexual roles in anal sex in the past 6 months (*n* = 1,525)	Receptive only	359 (23.5)
	Insertive only	541 (35.5)
	Versatile	625 (41.0)
Whether had group sex behavior in the past 6 months (*n* = 812)	None	602 (74.1)
	Seldom	184 (22.7)
	Sometimes	26 (3.2)
Whether had commercial sex with man in the past 6 months	No	1859 (89.3)
	Yes	222 (10.7)
Whether to use condoms when having heterosexual intercourse in the past 6 months	Always	839 (40.3)
	Sometimes	346 (16.7)
	Never	192 (9.2)
	Not having heterosexual intercourse	704 (33.8)
Whether have oral sex with man after drinking in the past 6 months	No	1,672 (80.3)
	Yes	409 (19.7)
Whether received HIV testing before entering the cohort	Yes	1,532 (73.6)
	No	549 (26.4)

**Table 2:** Univariate analysis between HIV positive and negative participants in Zhejiang (*n* = 2081).

Variable		Positive	Negative (*n* = 2045)	Statistics (t/χ^2^)	*p* value
		(*n* = 36)			
Observed person-years (PYs) Median (IQR)		0.745 (0.95)	0.64 (0.91)	0.634	0.526
Age Median (IQR)		28 (6)	27 (11)	0.390	0.696
Ethnicity					
	Han	33 (91.7)	1967 (96.1)	1.479	0.162
	Minority	3 (8.3)	78 (3.9)		
Local residence time				1.632	0.442
	Always	9 (25.0)	705 (34.5)		
	2 years and above	12 (33.3)	539 (26.4)		
	Less than 2 years	15 (41.7)	801 (39.1)		
Education level				7.971	0.184
	Bachelor’s degree and above	3 (8.3)	505 (24.7)		
	Junior college	10 (27.8)	510 (24.9)		
	Secondary school	3 (8.3)	213 (10.4)		
	High school	10 (27.8)	396 (19.4)		
	Junior high school	9 (25.0)	342 (16.7)		
	Elementary school and below	1 (2.8)	79 (3.9)		
Monthly income (yuan)				0.463	0.871
	10,000 and above	3 (8.3)	155 (7.6)		
	3,001–10,000	26 (72.2)	1,397 (68.3)		
	3,000 and below	7 (19.4)	493 (24.1)		
Marital status				2.328	0.310
	Divorced or widowed	1 (2.27)	133 (6.5)		
	Single	26 (72.2)	1,340 (65.5)		
	Married or cohabited	10 (27.8)	572 (28.0)		
Sexual orientation				0.007	0.883
	Homosexual	20 (55.6)	1,079 (52.8)		
	Bisexual	11 (30.6)	579 (28.3)		
	Heterosexual	1 (2.8)	88 (4.3)		
	Uncertain	4 (11.1)	299 (14.6)		
Whether had intercourse with man				0.443	0.506
	Yes	32 (88.9)	1736 (84.9)		
	No	4 (11.1)	309 (15.1)		
Number of male sexual partners in the past 6 months				Fisher	< 0.001
	None	2 (5.6)	164 (9.4)		
	1	7 (19.4)	811 (46.7)		
	2 to 5	19 (52.8)	656 (37.8)		
	6 and above	8 (22.2)	105 (6.0)		
Whether had HIV-positive sex partners in the past 6 months				Fisher	< 0.001
	Have an HIV-positive sex partner and have been receiving antiviral treatment	0	29 (1.8)		0.856
	Have an HIV-positive sex partner but have not been receiving antiviral treatment	0	8 (0.5)		
	Not clear	10 (33.3)	574 (36.5)		
	None	20 (66.7)	961 (61.1)		
Whether to use a condom during anal sex in the past 6 months				10.348	0.006
	Always	11 (37.9)	698 (46.7)		
	Sometimes	14 (48.3)	350 (23.4)		
	Never	4 (13.8)	448 (29.9)		
Sexual roles in anal intercourse				6.658	0.036
	Versatile	14 (48.3)	625 (41.0)		
	Receptive only	11 (37.9)	359 (23.5)		
	Insertive only	4 (13.8)	541 (35.5)		
Whether had group sex behavior in the past 6 months				0.024	0.288
	Sometimes	2 (8.3)	24 (3.0)		
	Seldom	5 (20.8)	179 (22.7)		
	None	17 (70.8)	585 (74.2)		
Whether had commercial sex with man in the past 6 months				0.000	1.000
	Yes	32 (88.9)	1827 (89.3)		
	No	4 (11.1)	218 (10.7)		
Whether to use condoms when having heterosexual sex in the past 6 months				1.388	0.708
	Not having heterosexual sex	12 (33.3)	692 (33.8)		
	Sometimes	7 (19.4)	339 (16.6)		
	Always	12 (33.3)	827 (40.4)		
	Never	5 (13.9)	187 (9.1)		
Have been diagnosed as STD in the past 6 months				0.428	0.684
	Syphilis	0 (0)	21 (1.0)		
	Other	1 (2.8)	43 (2.1)		
	None	35 (97.2)	1981 (96.9)		
Whether to use promotors during sex in the past 6 months				0.974	0.324
	Yes	7 (19.4)	256 (12.5)		
	No	29 (80.6)	1789 (87.5)		
Whether have oral sex with man after drinking in the past 6 months				0.007	0.415
	Yes	9 (25)	400 (19.6)		
	No	27 (75)	1,646 (80.4)		
Intervention				14.03	<0.001
	Yes	14 (38.9)	1,397 (68.3)		
	No	22 (61.1)	648 (31.7)		

### Univariate analysis between HIV positive and negative participants

The difference between HIV positive and negative MSM was shown in Table 3. A total of 1673.8 PYs of follow-up data were contributed by 2081 participants. 6,289 HIV antibody tests were performed in our cohort and 36 participants switched to HIV-positive during follow-up with overall HIV incidence being 2.15 per 100 PYs. The median follow-up time of the positive seroconversion participants was 0.745 years and 0.64 years for negative. A univariate analysis of the baseline socio-demographic characteristics showed that follow-up duration, age, ethnicity, local residence time, education level, monthly income, marital status had no statistical differences (*p* > 0.05). A univariate analysis of sexual behaviors between HIV-positive and HIV-negative individuals revealed statistically significant differences in the number of male sexual partners over the past 6 months, with HIV-positive MSM reporting a higher number of partners (Fisher *p* < 0.001). Consistent condom use during anal intercourse was more commonly observed in the HIV-negative group (*χ*^2^=10.348, *p* = 0.006). Additionally, during anal intercourse, individuals in the HIV-positive group were more likely to take the receptive role (χ^2^=6.658, *p* = 0.036).

**Table 3:** Multivariable analysis results of sexual behaviors between HIV positive and negative participants in Zhejiang.

Variable		Logistic regression		
		OR	95% CI	*p* value
Number of male sexual partners in the past 6 months	1	1.00	reference	
	2–5	2.46	0.48–12.58	0.208
	6 and above	4.20	1.67–10.59	0.013
Whether use condom during anal sex in the past 6 months	Always	1.00	reference	
	Sometimes	4.13	1.24–13.72	0.021
	Never	2.23	1.00–4.59	0.050
Sexual roles in anal sex in the past 6 months	Insertive only	1.00	reference	
	Versatile	3.81	1.18–12.25	0.025
	Receptive only	2.67	0.87–8.20	0.203
Intervention	No	1.00	reference	
	Yes	0.27	0.12–0.59	0.001

### Behavior difference between HIV positive and negative participants

Variables with *p*-value less than 0.05 in univariate analysis were included in multivariable logistic regression. The results showed that participants who had more than 6 partners in anal sexual behaviors are more vulnerable to HIV infection than those who had only one fixed partner (OR = 3.34, 95% CI: 1.28–8.71, *p* = 0.013). Compared to participants constantly use condoms during anal sex, never use condoms can obviously increase the risk of HIV infection (OR = 2.23, 95% CI: 1.00–4.59, *p* = 0.050) and sometimes use condoms in anal intercourse were also discovered as risk factor (OR = 4.13, 95% CI: 1.23–13.72, *p* = 0.021) (Table 3).

### Difference of sexual behaviors between student MSM and non-student MSM

The differences between student MSM and non-student MSM populations were shown in Supplementary Table 2. Student MSM participants were predominantly from Zhejiang Province (OR: 2.33, 95% CI: 1.54–3.54, *p* < 0.001) and were more likely to have received targeted interventions (OR: 2.10, 95% CI: 1.36–3.21, *p* = 0.001). They were also more likely to take receptive roles only (OR: 2.65, 95% CI: 1.62–4.08, *p* < 0.001) and both receptive and insertive roles (OR: 1.56, 95% CI: 1.05–2.32, *p* = 0.029) during anal intercourse. In contrast, they were less likely to engage in oral sex with men after drinking alcohol (OR: 0.32, 95% CI: 0.18–0.56, *p* < 0.001) and less likely to have ever take HIV testing (OR: 0.45, 95% CI: 0.31–0.66, *p* < 0.001).

### Risk factors of HIV infection among MSM

The number of sexual partners, sexual roles in anal intercourse and the use of condoms were included in multivariable Cox regression. We discovered that participants who had more than 6 partners in anal sexual intercourse were more likely to obtain HIV-seroconversion than who had one fixed partner (HR = 5.14, 95% CI: 1.67–10.59, *p* < 0.001). Compared to those who used condoms insistently in anal intercourse, sometime use condoms (HR: 4.11, 95% CI: 1.28–13.16, *p =* 0.017) and never use condoms (HR: 2.57, 95% CI: 1.16–5.66, *p* = 0.020) were more vulnerable to be infected by HIV. Versatile (HR: 5.30, 95% CI: 1.65–17.06, *p* = 0.005) and receptive only (HR: 3.23, 95% CI: 1.05–9.96, *p* = 0.042) were more likely to gain HIV infection than only insertive (Table 4).

**Table 4:** Cox regression results of sexual behaviors between HIV positive and negative participants in Zhejiang.

Variable		Cox regression		
		HR	95% CI	*p* value
Number of male sexual partners in the past 6 months	1	1.00	reference	
	2–5	2.94	0.58–14.86	0.191
	6 and above	5.14	1.67–10.59	0.000
Whether use condom during anal sex in the past 6 months	Always	1.00	reference	
	Sometimes	4.11	1.28–13.16	0.017
	Never	2.57	1.16–5.66	0.020
Sexual roles in anal sex in the past 6 months	Insertive only	1.00	reference	
	Versatile	5.30	1.65–17.06	0.005
	Receptive only	3.23	1.05–9.96	0.042
Intervention	No	1.00	reference	
	Yes	0.57	0.51–0.63	<0.001

## Discussion

Our study found that the HIV incidence density was 2.15 per 100 person-years (PYs) among the MSM population in Zhejiang, China. Having multiple sexual partners, inconsistent condom use, and assuming the receptive role during anal intercourse were identified as high-risk factors for HIV infection. Compared to non-student MSM, students MSM were less likely to have previous HIV testing and were more likely to take on the receptive role during anal intercourse.

Our research identifies several high-risk behavioral factors associated with HIV infection, including having multiple sexual partners, inconsistent condom use, and taking the receptive role during anal intercourse (RAI), significantly increase the likelihood of HIV transmission. This aligns with the findings of many previous studies (16, 17). Having multiple sexual partners increases the risk of HIV transmission due to a higher likelihood of encountering an HIV-positive individual, as well as greater exposure to the virus. The prevalence of multiple sexual partners in MSM populations may be linked to the relatively young age of subjects, which often makes it more difficult to establish stable, long-term relationships (16). In recent years, the use of pre-exposure prophylaxis (PrEP) with emtricitabine/tenofovir has contributed significantly to an increase in risky sexual behaviors, including inconsistent condom use and multiple sexual partners, within the MSM community due to that PrEP offering strong protection against HIV infection (18–20). This form of “prevention optimism,” while contributing to an increase in unprotected sexual behaviors, may also indirectly elevate the risk of bacterial STIs (21). Consistent condom use has long been recognized as one of the most effective measures for preventing HIV transmission during intercourse (22, 23). During condomless anal intercourse, the high tension of the anal sphincter and the fragile rectal mucosa, which may sustain microtears, make it more vulnerable to the invasion of HIV-contaminated bodily fluids, thereby facilitating viral transmission (24, 25). Inconsistent condom use among MSM is influenced by several factors. Perceived barriers—such as the belief that condoms reduce sexual pleasure, concerns about disrupting intimacy or trust with partners, and alcohol or drug use during sexual encounters—all contribute to this inconsistency (26). Alcohol or drug use impairs judgment and reduces the likelihood of condom use. A cross-sectional study of MSM living with HIV demonstrated that individuals with alcohol dependence had a higher likelihood of engaging in condomless anal intercourse due to impaired decision-making (27). Additionally, the pursuit of greater sexual satisfaction during anal intercourse often leads to a preference for condomless sex, which further contributes to inconsistent condom use. Our findings also highlight RAI as a significant risk factor for HIV infection. These results are consistent with prior studies showing that the risk of HIV transmission is several times higher for the receptive partner compared to the insertive partner during anal intercourse (28). From a physiological perspective, the rectal mucosa is thinner and more fragile than vaginal mucosa, making it more susceptible to microtears during anal intercourse (29). These microtears provide a direct pathway for HIV to enter the bloodstream, significantly increasing the risk of infection (24). Moreover, unlike the vagina, the rectum produces less natural lubrication, which increases friction and the likelihood of tissue damage, further enhancing the opportunity for viral exposure (30). During anal intercourse, the receptive partner is directly exposed to the insertive partner’s semen or penile secretions, which may contain a high concentration of HIV. When semen comes into contact with the rectal mucosa, especially in the presence of microtears, the virus can readily invade the mucosal tissue and infect target cells, significantly increasing the likelihood of HIV transmission (31).

The lower levels of previous HIV testing among student MSM in our study highlights a significant concern for HIV prevention efforts in this group. HIV testing is a critical tool in achieving global HIV prevention and treatment goals, as it allows individuals to determine their HIV status and access timely care (32). However, it is estimated that nearly 6 million people living with HIV were unaware of their HIV status in 2021 (33). Among MSM populations, including students, barriers to HIV testing persist and need to be addressed to reduce the risk of HIV transmission. Several sociocultural factors contribute to the reluctance to undergo HIV testing in the student MSM population. HIV/AIDS-related stigma remains prevalent within many student communities, leading to resistance toward testing due to fears of being outed regarding both sexual orientation and health status (34, 35). In addition, the psychological fear of receiving a positive HIV diagnosis can deter students from seeking testing, as they may lack sufficient mental health support or counseling services to cope with such a result (36). Behavioral factors, such as peer influence, also play a significant role in testing behavior (37). Students are often influenced by the attitudes and behaviors of those around them, and if HIV testing is not prioritized within their social network, individuals may be less inclined to seek testing themselves. Furthermore, the availability of HIV testing services on college campuses may be limited, reducing students’ access to convenient testing options and further discouraging them from undergoing HIV testing (38). Another key finding of this study was that student MSM were more likely to assume the receptive role during anal intercourse, which has been associated with an increased risk of HIV acquisition. The physiological vulnerability of the receptive partner during anal intercourse, especially in men, is well-documented, as the male anus is more susceptible to injury and thus a more direct pathway for HIV transmission (39). Additionally, sexual roles in male–male relationships often carry psychological and sociocultural connotations, with the receptive role being linked to more feminine gender traits, while the insertive role is commonly associated with masculinity (40). These gendered perceptions of sexual roles may influence sexual behavior and contribute to the higher risks faced by student MSM, particularly those engaging in receptive anal intercourse. Given the high HIV infection rates among receptive partners and the low levels of HIV testing among student MSM, there is an urgent need for targeted health education and interventions on campuses.

There are several limitations to our study. First, the average follow-up duration per participant was relatively short, which may hinder our ability to capture enough HIV cases and obtain more robust findings. Second, social desirability bias and recall bias may arise from analyses based on self-reported data. Third, selection bias could result from our non-randomized sampling method, limiting the generalizability of our findings to the entire MSM population and consequently constraining our conclusions. The future research could benefit from longer follow-up periods and a more representative study population.

## Conclusion

Our study revealed that the HIV incidence density among the MSM population in Zhejiang, China, was 2.15 per 100 person-years. Key high-risk factors for HIV infection included having multiple sexual partners, inconsistent condom use, and assuming the receptive role during anal intercourse. Notably, students MSM were less likely to have undergone previous HIV testing compared to their non-student MSM counterparts and were more inclined to take on the receptive role during anal intercourse. Increased focus and interventions are essential to encourage safer sexual practices and mitigate HIV transmission within this population.

## Data Availability

The raw data supporting the conclusions of this article will be made available by the authors, without undue reservation.
