# Supplementary material for: Incidence of HIV infection and associated factors among men who have sex with men in Zhejiang, China: a cohort study
Source: Front Public Health. 2025 Apr 28;13:1551612. doi: 10.3389/fpubh.2025.1551612 (PMC12066761; doi:10.3389/fpubh.2025.1551612)
Supplement: Supplementary file 1 [file Table_1.docx]

| **Supplementary Table 1.** Basic characteristics of student MSM and non-student MSM in Zhejiang (n=2081) | | | | | | |  |  |  |
| --- | --- | --- | --- | --- | --- | --- | --- | --- | --- |
| Variables |  | Category |  |  | n (%) | | χ2 | *p*-value |  |
|  |  |  |  |  | Student MSM (n=214) | Non-student MSM (n=1867) |  |  |  |
| Age | |  | |  |  |  | 368.49 | < 0.001 |  |
|  | | 18-24 | |  | 201 (94.0) | 523(28.1) |  |  |  |
|  | | 25-34 | |  | 13 (6.0) | 827(44.3) |  |  |  |
|  | | 35-44 | |  | 0 (0) | 333(17.8) |  |  |  |
|  | | ≥45 | |  | 0 (0) | 184(9.8) |  |  |  |
| Ethnicity | |  |  |  |  |  | 0.015 | 0.902 |  |
|  |  | Han | |  | 206 (96.2) | 1794 (96.0) |  |  |  |
|  |  | Minority | |  | 8 (3.7) | 73 (4.0) |  |  |  |
| Local residence time | |  |  |  |  |  | 8.35 | 0.015 |  |
|  |  | Always | |  | 82 (38.3) | 632 (33.8) |  |  |  |
|  |  | 2 years and above | |  | 39 (18.3) | 512 (27.5) |  |  |  |
|  |  | Less than 2 years | |  | 93 (43.4) | 723 (38.7) |  |  |  |
| Education level | |  |  |  |  |  | 92.92 | < 0.001 |  |
|  |  | Postgraduate and above | |  | 17 (7.9) | 46 (2.4) |  |  |  |
|  |  | Undergraduate (College) | |  | 148 (69.2) | 817 (43.8) |  |  |  |
|  | | High school (secondary school) | |  | 45 (21.1) | 577 (30.9) |  |  |  |
|  |  | Junior high school | |  | 3 (1.4) | 348 (18.7) |  |  |  |
|  |  | Elementary school and below | |  | 1 (0.4) | 79 (4.2) |  |  |  |
| Monthly income(yuan) | No income |  | | 107 (50.0) | 92 (4.9) | 755.445 | < 0.001 |  |  |
|  | |  | | ≤1500 |  | 47 (22.0) | 34 (1.8) |  |  |
|  |  | 1501-3000 | |  | 38 (17.8) | 182 (9.8) |  |  |  |
|  |  | 3001-5000 | |  | 13 (6.0) | 691 (37.2) |  |  |  |
|  |  | 5001-10000 | |  | 8 (3.8) | 711 (38.0) |  |  |  |
|  |  | 10001-50000 | |  | 1 (0.4) | 136 (7.2) |  |  |  |
|  |  | Above 50000 | |  | 0 (0.0) | 21 (1.1) |  |  |  |
| Marital status |  |  | |  |  |  | 121.483 | < 0.001 |  |
|  | | Single |  | 213 (99.5) | 1153 (61.7) |  |  |  |  |
|  | |  | | Married or cohabited |  | 1 (0.5) | 581(31.2) |  |  |
|  |  | Divorced or widowed | |  | 0 (0.0) | 133 (7.1) |  |  |  |
| Sexual inclination |  |  | |  |  |  | 3.163 | 0.367 |  |
|  | |  | Homosexual |  | 124 (57.9) | 975 (52.3) |  |  |  |
|  |  | Bisexual | |  | 51 (23.9) | 539 (28.9) |  |  |  |
|  |  | heterosexual | |  | 10 (4.7) | 79 (4.2) |  |  |  |
|  |  | Uncertain | |  | 29 (13.5) | 274 (14.6) |  |  |  |
| Whether had anal or oral intercourse with man |  |  | |  |  |  | 2.106 | 0.147 |  |
|  | |  | Yes |  | 189 (88.4) | 1579 (84.5) |  |  |  |
|  |  | No | |  | 25 (11.6) | 288 (15.5) |  |  |  |
| Number of male sexual partners in the past 6 months (n=1768) |  |  | |  |  |  | 4.911 | 0.297 |  |
| Number of male sexual partners in the past 6 months (n=1768) | | None |  | 12 (6.3) | 154 (9.8) |  |  |  |  |
|  |  |  | | 1 |  | 84 (44.4) | 734 (46.5) |  |  |
|  |  | 2 to 5 | |  | 77 (40.7) | 600 (37.9) |  |  |  |
|  |  | 6 to 9 | |  | 10 (5.4) | 51 (3.3) |  |  |  |
|  |  | 10 and above | |  | 6 (3.2) | 40 (2.5) |  |  |  |
| Whether had HIV-positive sex partners in the past 6 months (n=1654) |  |  | |  |  |  | 9.702 | 0.016 |  |
|  | |  | None |  |  | 97 (54.9) | 884 (62.0) |  |  |
|  |  | Have an HIV-positive sex partner and have been receiving antiviral treatment |  | 1 (0.5) | 28 (22.0) |  |  |  |  |
|  |  |  | |  |  |  |  |  |  |
|  |  | Have an HIV-positive sex partner but have not been receiving antiviral treatment | |  | 3 (1.7) | 5 (0.3) |  |  |  |
|  |  | Not clear | |  | 76 (42.9) | 508 (35.7) |  |  |  |
| Whether to use a condom during anal intercourse in the past 6 months (n=1602) |  |  | |  |  |  | 13.461 | 0.004 |  |
|  | | Always |  |  | 88 (49.8) | 878 (61.7) |  |  |  |
|  |  | Sometimes | |  | 63 (35.6) | 415 (29.2) |  |  |  |
|  |  | Never | |  | 10 (5.6) | 71 (4.9) |  |  |  |
|  |  | No anal intercourse | |  | 16 (9.0) | 61 (4.2) |  |  |  |
| Sexual roles in anal sex months(n=1525) |  |  | |  |  |  | 23.98 | < 0.001 |  |
|  | | Receptive only |  |  | 60 (37.2) | 299 (21.9) |  |  |  |
|  |  | Insertive only | |  | 35 (21.7) | 506 (37.0) |  |  |  |
|  |  | Versatile | |  | 66 (40.9) | 559 (40.9) |  |  |  |
| Whether had group sex behavior in the past 6 months (n=812) |  |  | |  |  |  | 3.324 | 0.19 |  |
|  | |  | None |  | 77 (81.0) | 525 (73.3) |  |  |  |
|  |  | Seldom | |  | 17 (17.9) | 167 (23.2) |  |  |  |
|  |  | Sometimes | |  | 1 (1.1) | 25 (3.5) |  |  |  |
| Whether had commercial sex with man in the past 6 months |  |  | |  |  |  | 8.996 | 0.003 |  |
|  | |  | No |  | 204 (95.3) | 1655 (88.6) |  |  |  |
|  |  | Yes | |  | 10 (4.7) | 212 (11.4) |  |  |  |
| Whether to use condoms when having heterosexual intercourse in the past 6 months |  |  | |  |  |  | 44.78 | < 0.001 |  |
|  | | Always |  |  | 56 (26.2) |  |  |  |  |
|  |  |  |  |  |  | 783 (41.9) |  |  |  |
|  |  | Sometimes | |  | 28 (13.0) | 318 (17.0) |  |  |  |
|  |  | Never | |  | 14 (6.6) | 178 (9.6) |  |  |  |
|  |  | Not having heterosexual intercourse | |  | 116 (54.2) | 588 (31.5) |  |  |  |
| Whether have oral sex with man after drinking in the past 6 months |  |  | |  |  |  | 8.507 | 0.004 |  |
|  | |  | No |  | 188 (87.8) | 1484 (79.5) |  |  |  |
|  |  | Yes | |  | 26 (12.2) | 383 (20.5) |  |  |  |
| Whether received HIV testing before entering the cohort |  |  | |  |  |  | 21.849 | < 0.001 |  |
|  | |  | Yes |  | 129 (60.3) | 1403 (75.2) |  |  |  |
|  |  | No | |  | 85 (39.7) | 464 (24.8) |  |  |  |

| **Supplementary Table 2.** Multivariate analysis results of behaviors between students MSM and non-student MSM in Zhejiang province (n=788) | | | | |
| --- | --- | --- | --- | --- |
| Variable |  | OR | 95%CI | P value |
| Domicile place |  |  |  |  |
|  | Other provinces | 1.00 | reference |  |
|  | Zhejiang Province | 2.33 | 1.54-3.54 | <0.001 |
| Number of male sexual partners in the past 6 months |  |  |  |  |
|  | 1 | 1.00 | reference |  |
|  | 2 and above | 1.17 | 0.82-1.67 | 0.383 |
| Whether use condom during anal sex in the past 6 months |  |  |  |  |
|  | Always | 1.00 | reference |  |
|  | Sometimes | 1.26 | 0.60-2.64 | 0.536 |
|  | Never | 0.92 | 0.43-1.94 | 0.890 |
| Sexual roles in anal sex in the past 6 months | |  |  |  |
|  | Insertive only | 1.00 | reference |  |
|  | Versatile | 1.56 | 1.05-2.32 | 0.029 |
|  | Receptive only | 2.65 | 1.62-4.08 | <0.001 |
| Whether to use promotors during sex in the past 6 months |  |  |  |  |
|  | No | 1.00 | reference |  |
|  | Yes | 1.20 | 0.75-1.94 | 0.439 |
| Whether to have oral sex with man after drinking in the past 6 months |  |  |  |  |
|  | No | 1.00 | reference |  |
|  | Yes | 0.32 | 0.18-0.56 | <0.001 |
| Intervention |  |  |  |  |
|  | No | 1.00 | reference |  |
|  | Yes | 2.10 | 1.36-3.21 | 0.001 |
| Whether having been tested for HIV in the past |  |  |  |  |
|  | No | 1.00 | reference |  |
|  | Yes | 0.45 | 0.31-0.66 | <0.001 |
| Duration of residence |  |  |  |  |
|  | Do not reside locally | 1.00 | reference |  |
|  | Less than 6 months | 0.22 | 0.11-0.44 | <0.001 |
|  | 6 months to 2 years | 0.80 | 0.40-1.58 | 0.634 |
|  | Above 2 years | 1.05 | 0.65-1.63 | 0.836 |
|  | Always reside locally | 1.42 | 0.85-2.38 | 0.178 |
